# MDM2/MDMX inhibition by Sulanemadlin synergizes with anti-Programmed Death 1 immunotherapy in wild-type p53 tumors

**DOI:** 10.1016/j.isci.2024.109862

**Published:** 2024-05-06

**Authors:** Katrine Ingelshed, Marit M. Melssen, Pavitra Kannan, Arun Chandramohan, Anthony W. Partridge, Long Jiang, Fredrik Wermeling, David P. Lane, Marika Nestor, Diana Spiegelberg

**Affiliations:** 1 Department of Microbiology, Tumor and Cell Biology, Karolinska Institutet, 17177 Stockholm, Sweden; 2 Department of Immunology, Genetics and Pathology, Uppsala University, 75185 Uppsala, Sweden; 3 MSD International Singapore, Singapore 138665, Singapore; 4 Division of Rheumatology, Department of Medicine Solna, Karolinska University Hospital and Karolinska Institutet, 17177 Stockholm, Sweden; 5 Center for Molecular Medicine, 17176 Stockholm, Sweden; 6 Department of Surgical Sciences, Uppsala University, 75185 Uppsala, Sweden

**Keywords:** microenvironment, Molecular biology, Immunology, cancer

## Abstract

Immunotherapy has revolutionized cancer treatment but its efficacy depends on a robust immune response in the tumor. Silencing of the tumor suppressor p53 is common in tumors and can affect the recruitment and activation of different immune cells, leading to immune evasion and poor therapy response. We found that the p53 activating stapled peptide MDM2/MDMX inhibitor Sulanemadlin (ALRN-6924) inhibited p53 wild-type cancer cell growth *in vitro* and *in vivo*. In mice carrying p53 wild-type CT26.WT tumors, monotherapy with the PD-1 inhibitor DX400 or Sulanemadlin delayed tumor doubling time by 50% and 37%, respectively, while combination therapy decreased tumor doubling time by 93% leading to an increased median survival time. Sulanemadlin treatment led to increased immunogenicity and combination treatment with PD-1 inhibition resulted in an increased tumor infiltration of lymphocytes. This combination treatment strategy could potentially turn partial responders into responders of immunotherapy, expanding the patient target group for PD-1-targeting immunotherapy.

## Highlights

• MDM2/MDMX inhibition by Sulanemadlin reduces cell growth dependent on p53 status
• p53 activation by Sulanemadlin leads to increased immunogenicity marker expression
• Sulanemadlin + anti-PD-1 immunotherapy increases lymphocyte infiltration
• Sulanemadlin synergizes with anti-PD-1 immunotherapy increasing overall survival

## Graphical abstract

**Figure:**
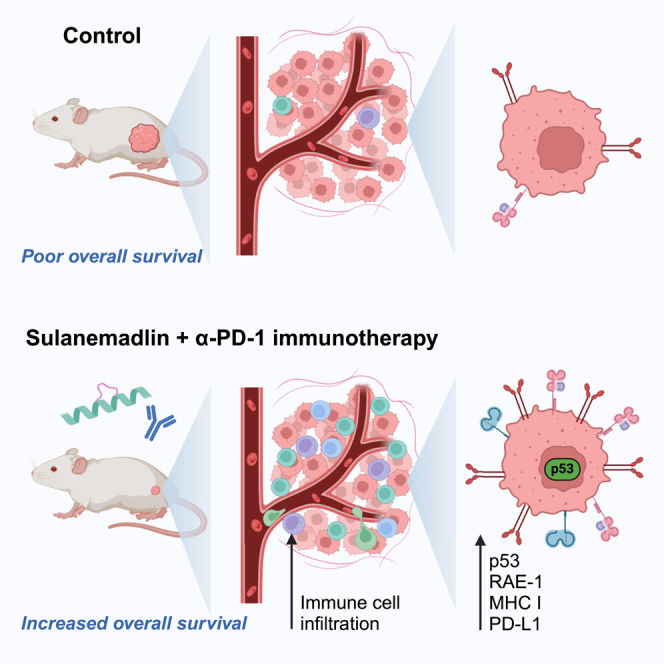

## Introduction

The tumor suppressor protein p53 is a transcription factor that regulates various crucial cellular mechanisms such as the control of cell-cycle arrest, apoptosis, and senescence.^1^ The expression of p53 is directly regulated by its interaction with the E3 ubiquitin ligase MDM2 and its homolog MDMX (also commonly referred to as MDM4), in a heterodimer complex.^2^^,^^3^ In healthy, non-stressed cells, p53 is constantly produced but degraded by MDM2.^2^ The E3 ubiquitin ligase activity of MDM2 is partially dependent on MDMX binding to ubiquitin-conjugating enzymes.^4^ Hence, both proteins in complex are needed to efficiently regulate p53 activity.^5^

Dysfunctional regulation of p53 is involved in nearly all tumors. p53 can be silenced either through direct mutations, occurring in about 50% of all tumors, or through upstream alterations leading to an inability in regulating MDM2 expression and increased MDM2/MDMX activity and inhibition of p53.^6^^,^^7^^,^^8^^,^^9^^,^^10^ Consequently, cancer patients with wild-type (wt) p53 tumors could potentially benefit from reactivation of normal p53 function. Several investigational new drugs are being developed for this purpose, mostly focused on the inhibition of MDM2.^11^ The first MDM2 inhibitors developed were the Nutlin compounds.^12^ These small molecule inhibitors of MDM2 were later further evolved and Idasanutlin (RG-7388) is currently being evaluated in clinical trials (NCT04029688, NCT02633059, and NCT04589845).^13^ Other potent small molecule MDM2 inhibitors have also been developed more recently, among these are Navtemadlin (AMG 232/KRT 232) and Siremadlin (HDM201), both actively investigated in clinical trials (NCT03217266 and NCT05447663 among others).^14^^,^^15^ Stapled peptides that inhibit MDM2 and MDMX have also proved efficient in activating the p53 pathway.^16^^,^^17^^,^^18^ These peptides mimic the p53 motif binding to the MDM2-MDMX complex.^19^^,^^20^^,^^21^ Hydrocarbon staples have further been added to the peptide to lock the peptide in a specific conformation, increasing its stability and protecting it from degradation by proteases.^22^ This gives stapled peptides a possible advantage over small molecule MDM2 inhibitors which only inhibit MDM2 and are rapidly degraded.^18^^,^^22^^,^^23^ The only MDM2/MDMX stapled peptide inhibitor currently in clinical trials is Sulanemadlin (ALRN-6924) (NCT03725436).

MDM2 inhibition is also increasingly gathering interest as a potential effector of immunotherapy response.^24^^,^^25^^,^^26^ As a result of the discovery of the immune checkpoints CTLA4 and the PD-1/PD-L1 axis and the subsequent development of monoclonal antibodies targeting these immune checkpoints, patient survival has improved in several cancer types.^27^ Activated cytotoxic T cells are an important part of the defense against tumors, but chronic reactivation by tumor cells or chronic viral infections lead to exhaustion.^28^ One feature of T cell exhaustion involves increased expression of PD-1, leading to T cell inhibition when bound to its ligand PD-L1 or PD-L2.^29^ Tumor cells and myeloid cells in the tumor microenvironment often express PD-L1, thereby dampening T cell function. An upregulated expression of PD-L1 in tumor cells may therefore contribute to tumor progression even if tumor eradicating leukocytes are infiltrating the tumor.^30^ Since the success of the PD-1 blockade depends on immune cell infiltration in the tumors, the majority of patients still experience limited response to the treatment.^31^ There is therefore a significant interest in enhancing the immunogenicity of the tumor as a combination treatment strategy. To date, several studies have suggested that inhibition of MDM2 may in fact enhance the immunological response.^25^^,^^26^^,^^32^^,^^33^ It is however not clear how immunogenicity is affected by MDM2 and MDMX inhibition. So far, only small molecule MDM2 inhibitors like Navtemadlin in combination with PD-1 and PD-L1 blocking antibodies are being investigated in clinical trials (NCT03787602and NCT05705466). Sulanemadlin, the only dual MDM2/MDMX inhibitor in clinical trials, has to our knowledge not been evaluated in combination with immunotherapy.

The CT26.WT colon carcinoma syngeneic mouse model presents a unique challenge due to its partial responsiveness to anti-PD-1 treatment.^34^ CT26.WT cells are known to exhibit intermediate immunogenicity, meaning they are less likely to be recognized and targeted by the immune system compared to highly immunogenic cells. Low to intermediate immunogenicity can make it difficult to observe significant changes in immune responses or therapeutic effects, particularly when testing interventions aimed at modulating immune activity. However, this limited response resembles the complexities observed in the clinical context.

In the present study, we have investigated the effects of MDM2 and MDM2/MDMX inhibition as monotherapy and in combination with anti-PD-1 immunotherapy in the p53-wildtype (CT26.WT) syngeneic mouse model. We report a synergistic tumor reducing effect when the MDM2/MDMX inhibitor Sulanemadlin is combined with anti-PD-1 therapy with the monoclonal antibody DX400. The immunogenicity of the tumors and infiltration of T cells and natural killer cells into the tumors was increased by the combination treatment and correlated to a decreased tumor size. Hence, our data suggest that patients that are only partial responders to PD-1 blockade may benefit from additional treatment with Sulanemadlin.

## Results

### Stapled peptide Sulanemadlin and small molecule MDM2 inhibitors Siremadlin and Navtemadlin efficiently inhibit cell growth p53 dependently *in vitro*

**Figure 1:**
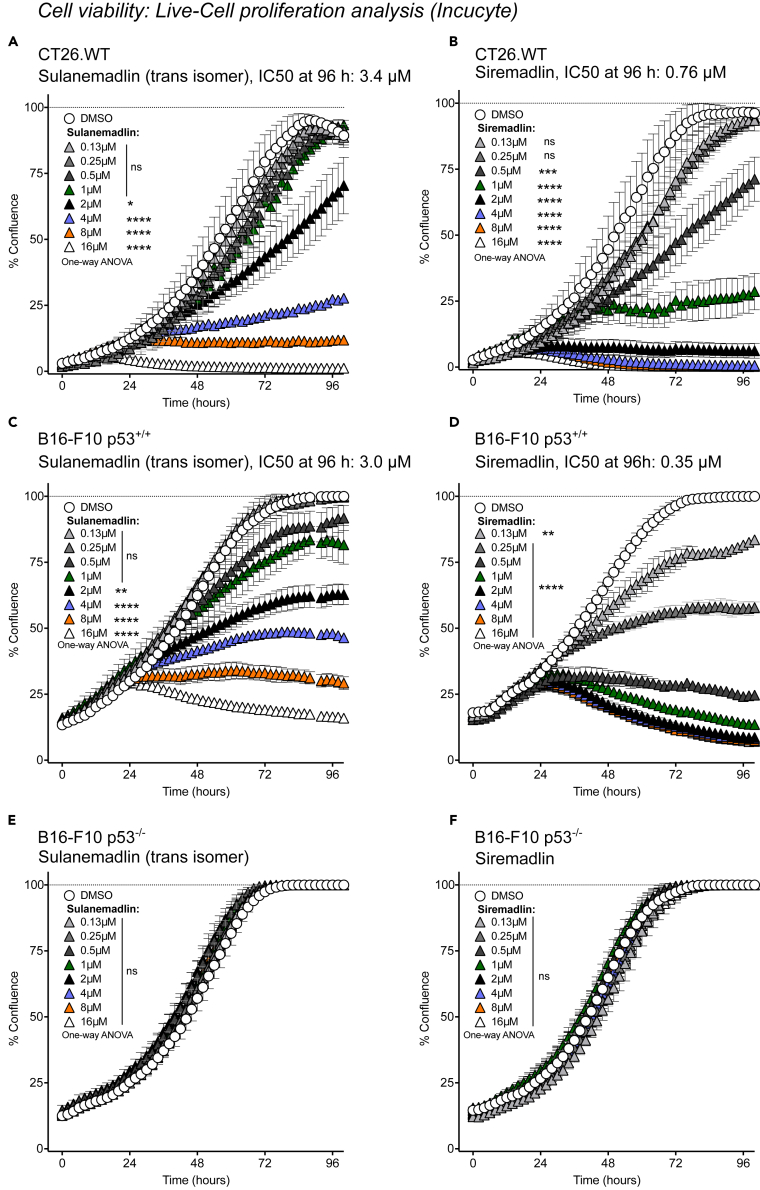
Stapled peptide Sulanemadlin and small molecule MDM2 inhibitors Siremadlin and Navtemadlin efficiently inhibit cell growth p53 dependently *in vitro* (A–F) CT26.WT murine colon carcinoma cells, B16-F10 p53^+/+^ and B16-F10 p53^−/−^ murine malignant melanoma cells were treated with indicated concentrations of Sulanemadlin and Siremadlin and cell growth was monitored in the Incucyte S3 live cell imaging system. Proliferation was calculated as % of confluence over time. IC_50_ values were normalized to DMSO control and calculated after 96 h treatment. Representative data from at least two experiments performed in triplicates and a mean IC50 from all performed experiments are shown. ∗*p* < 0.05; ∗∗*p* < 0.01; ∗∗∗*p* < 0.001; ∗∗∗∗*p* < 0.0001. One-way ANOVA, (data are represented as mean with SD). See also Figures S1 and S2.

We set out to determine the *in vitro* potency of the two new generation small molecule MDM2 inhibitors Siremadlin and Navtemadlin as well as the stapled peptide Sulanemadlin, which uniquely inhibits both MDM2 and MDMX. We first treated the murine colon carcinoma cell line CT26.WT with Sulanemadlin, Siremadlin, and Navtemadlin at concentrations ranging from 0.13–16 μmol/L. Cells were imaged every 2 h during treatment using the IncuCyte S3 live cell imaging system. Cell confluency and IC_50_ values were calculated after 96 h. All three treatments resulted in reduced growth, with only debris or debris together with a few elongated cells at the highest concentrations after 96 h of treatment (Figure S1). The *trans*-isomer of the stapled peptide Sulanemadlin, used in clinical trials, had an IC_50_ of 3.4 μmol/L (Figure 1A) while the mixed isomer form (*cis* and *trans*) of Sulanemadlin required slightly higher concentrations to reduce cell growth to the same extent, with an IC_50_ of 4.7 μmol/L (Figure S2A). The inactive version of Sulanemadlin, referred to as the non-binder, had no effect on the cell growth even at the highest concentration (Figure S2B). The small molecule inhibitors inhibited 50% of the CT26.WT cell growth already at 0.76 μmol/L (Siremadlin) and 2.2 μmol/L (Navtemadlin) (Figures 1B and S2C). To affirm that this effect on cell growth is dependent on p53 activation, we next treated and compared the cell growth of the murine cell line B16-F10 carrying wt p53 and the B16-F10 cell line with a p53 deletion (B16-F10 p53^−/−^). The IC_50_ concentrations of all three compounds in the B16-F10 p53^+/+^ cells were similar to the IC_50_ concentrations in the CT26.WT cells, with 50% of cell growth inhibited at 3 μmol/L (Sulanemadlin *trans* isomer), 0.35 μmol/L (Siremadlin), and 2 μmol/L (Navtemadlin) (Figures 1C, 1D, and S2D). However, there was no significant difference in the growth of treated B16-F10 p53^−/−^ cells, confirming that the cell growth is indeed inhibited by p53 activity (Figures 1E, 1F, and S2E). Since it has previously been suggested that the efficiency of MDM2 inhibitors can vary with species,^35^ we also treated the human osteosarcoma cell line SJSA-1, harboring wt p53, with Sulanemadlin, Siremadlin, and Navtemadlin. Indeed, 50% of growth of SJSA-1 cells was efficiently inhibited at very low concentrations 0.80 μmol/L (Sulanemadlin *trans* isomer), 0.22 μmol/L (Siremadlin), and 0.28 μmol/L (Navtemadlin) (Figures S2F–S2H). This demonstrates that Sulanemadlin, Siremadlin, and Navtemadlin all efficiently inhibit cell growth, in both murine and human cells. We further show that this growth arrest is dependent on the expression of wild-type p53.

### Activation of p53 by inhibition of MDM2/MDMX with Sulanemadlin leads to increased expression of immunogenicity markers

Next, we wanted to examine whether activation of p53 by the dual MDM2-MDMX inhibitor Sulanemadlin could alter the expression of proteins relevant to PD-1 immunotherapy. We treated the murine colorectal cancer cell line CT26.WT with Sulanemadlin (*trans* isomer) for 48 h when the first effect on cell confluency was observed (Figure 1A), at a concentration close to the IC_50_ concentration of Sulanemadlin, 4 μmol/L.

**Figure 2:**
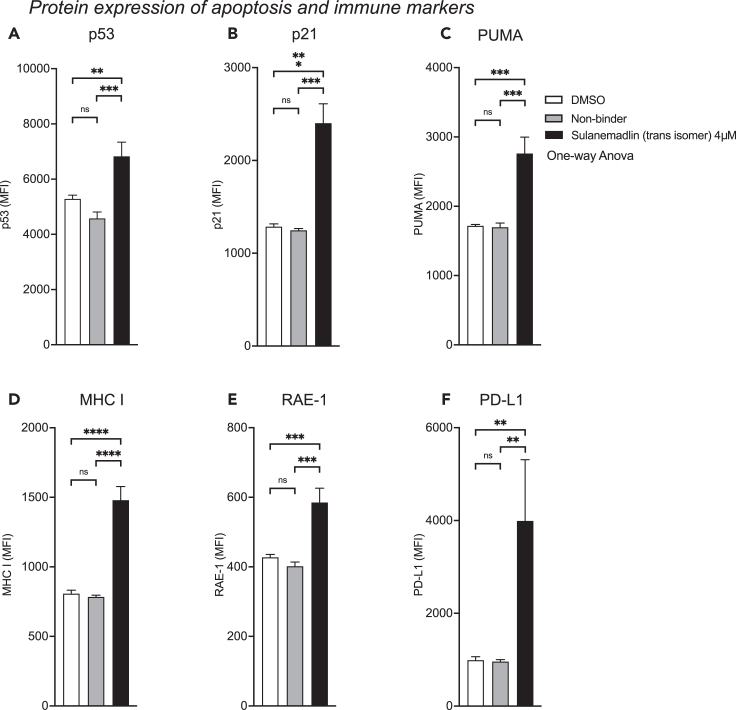
Activation of p53 by inhibition of MDM2/MDMX with Sulanemadlin leads to increased expression of immunogenicity markers CT26.WT cells were treated with 4 μmol/L Sulanemadlin for 48 h and flow cytometry analysis of protein expression determined the median fluorescence intensity (MFI) of (A) p53, (B) p21, (C) PUMA, (D) MHC I, (E) RAE-1, (F) PD-L1. Gating according to FMO controls excluded debris and only included single live cells. Representative data from two separate experiments performed in triplicates. ∗∗*p* < 0.01; ∗∗∗*p* < 0.001; ∗∗∗∗*p* < 0.0001. One-way ANOVA, (data are represented as mean with SD). See also Figures S3 and S4.

The protein analysis revealed that Sulanemadlin significantly increased the expression of p53, as expected, consistent with the compound’s reported mechanism of action. Furthermore, Sulanemadlin treatment increased the cyclin-dependent kinase inhibitor p21, responsible for regulating cell-cycle arrest, and the pro-apoptotic protein PUMA (Figures 2A–2C), both downstream targets of p53, confirming the p53 activating effects of the compound.

However, the immune system can only target tumor cells that are recognized to be defective. Cytotoxic CD8^+^ T cells require the tumor to present tumor antigen in MHC class I molecules for recognition and activation.^36^ Reduced MHC class I expression helps the tumor cells evade CD8^+^ T cell activity.^37^ NK cells, on the other hand, can recognize cancer cells that have reduced MHC class I expression as well as stress-induced ligands like RAE-1.^38^^,^^39^ The protein analysis showed that Sulanemadlin significantly induced the expression of MHC class I molecules, the NK cell activating ligand RAE-1 as well as PD-L1 (Figures 2D–2F), while treatment with an unspecific stapled peptide (non-binder) did not alter the expression of any of the analyzed proteins (Figures 2A–2F).

In line with our previously reported data on the p53 dependent upregulation of p53 downstream targets by Navtemadlin in B16-F10 cells,^40^ we also detected an upregulation of PD-L1 and MHC I, normally not expressed by B16-F10 cells, in B16-F10 p53^+/+^ cells treated with Sulanemadlin (Figure S3A) and Navtemadlin (Figure S3B). Navtemadlin treatment also induced the expression of RAE-1 in B16-F10^+/+^ cells (Figure S3B). None of these proteins were upregulated upon treatment in the B16-F10 p53^−/−^ cells (Figures S3A and S3B).

We further examined the upregulation of p53 induced by Siremadlin and Navtemadlin in comparison to Sulanemadlin in CT26.WT cells (Figure S3C). Notably, only Sulanemadlin showed a significant increase in p53 levels at the respective IC50 concentrations. Interestingly, the IC50 concentrations of all drugs induced the expression of the p53 downstream target PUMA. In addition, an increase in the immunogenicity marker MHC I was observed 48 h after exposure to the inhibitors. PD-L1 was however only increased after treatment with Siremadlin in the CT26.WT cells. To investigate further the lack of p53 upregulation in CT26-WT at IC_50_ for Siremadlin and Navtemadlin, we examined higher drug concentrations (three times the IC_50_ concentration) shown in Figures S3D and S4. Here, as expected, increasing concentration resulted in a concentration-dependent increase in p53 expression. This observation seems to be CT26.WT specific as IC_50_ drug concentrations were able to increase p53 in B16-F10 cells (Figures S3F and S4).

We conclude that Sulanemadlin treatment increases the protein expression of MHC I and RAE-1, proteins important for immune cell detection of tumor cells. The mechanisms behind PD-L1 expression are not fully understood yet. However, PD-L1 expression has been indirectly correlated to p53 expression.^41^^,^^42^ As PD-L1 is upregulated by p53 therapy, this treatment strategy may benefit from being combined with an anti-PD-1 treatment in an *in vivo* setting.

### *In vivo* treatment with the stapled peptide Sulanemadlin leads to increased survival which is further enhanced by combination with anti-PD-1 immunotherapy

**Figure 3:**
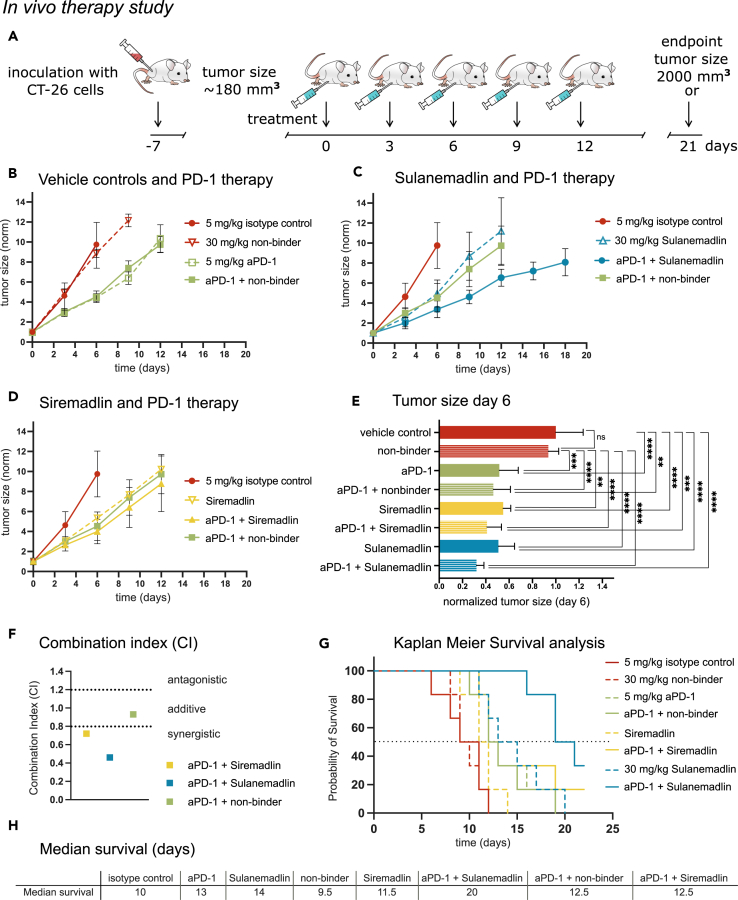
*In vivo* treatment with the stapled peptide Sulenemadlin leads to increased survival which is further enhanced by combination with anti-PD-1 immunotherapy (A) Overview of the treatment schedule. BALB/c mice carrying CT26.WT tumors were treated with isotype control (5 mg/kg), non-binder (30 mg/kg), Sulanemadlin (30 mg/kg, mixed isomer), and Siremadlin (30 mg/kg) as well as combination therapy of anti-PD-1 immunotherapy (aPD-1) (5 mg/kg) in combination with non-binder (30 mg/kg), anti-PD-1 (5 mg/kg) in combination with Sulanemadlin (30 mg/kg, mixed isomer), and anti-PD-1 (5 mg/kg) in combination with Siremadlin (30 mg/kg). The endpoint was defined by tumor size reaching 2,000 mm^3^ or 21 days after treatment started. (B–D) Tumor sizes were measured every third day and normalized to the tumor size at treatment start (day 0, average size 180 mm^3^), plotted as mean with SD. (E) Tumor sizes in the monotherapies and the control treated groups as well as the combination treated groups at day 6. ∗∗*p* < 0.01; ∗∗∗*p* < 0.001; ∗∗∗∗*p* < 0.0001. One-way ANOVA, (data are represented as mean with SD). (F) Combination Index (CI) for anti-PD-1 and Siremadlin, anti-PD-1 and Sulanemadlin, and anti-PD-1 and non-binder. (G) Kaplan Meier survival analysis of the *in vivo* study. Survival was plotted as % of mice still alive over time. Dashed line represents 50% survival. (H) Median survival time in days after treatment start (50% survival) of the mice in the different treatment groups. See also Figure S5 and Table S1.

We further evaluated the efficacy of the stapled peptide Sulanemadlin (mixed isomer), Siremadlin, and anti-PD-1 (DX400) monotherapy and their combination using a syngeneic tumor model with subcutaneous CT26.WT colon tumors. To ensure the sensitivity and specificity of the used compounds and to exclude any non-specific effects, several control treatments were used in the experimental setup. The experimental setup and treatment schedule is summarized in Figure 3A. Treatment with 30 mg/kg non-binder or an isotype control antibody (5 mg/kg) had no significant effect on tumor growth or overall survival of the mice (Figures 3B–3H). However, monotherapy with 5 mg/kg anti-PD-1 immunotherapy, 30 mg/kg Siremadlin, and 30 mg/kg Sulanemadlin significantly delayed tumor growth with a tumor doubling time of 1.83 days in the control group to 2.72, 2.48, and 2.6 days, respectively (Figures 3B–3E, S5, and Table S1). Combination treatment of 5 mg/kg anti-PD-1 therapy and 30 mg/kg Siremadlin as well as 5 mg/kg anti-PD-1 therapy and 30 mg/kg Sulanemadlin resulted in a further prolongation of tumor doubling times to 2.97 and 3.42 days, respectively (Table S1).

One week after treatment start at day 6, the first animal (control group) reached the study endpoint (tumor size >2 cm^3^). There was a significant difference in tumor size of the monotherapies Sulanemadlin, Siremadlin, and PD-1 immunotherapy, compared to both control groups (vehicle control and non-binder). Mice in the combination groups Sulanemadlin + PD-1 immunotherapy and Siremadlin + immunotherapy had a statistically significant smaller tumor size than mice in the vehicle control or non-binder group (Figure 3E). Furthermore, the combination effects of the treatment groups were assessed at day 6 according to the Chou-Talalay method for drug combinations,^43^ with a combination index (CI) of < 0.8 indicating synergy (Figure 3F). As previously described, combining anti-PD-1 immunotherapy with the non-specific stapled peptide (non-binder) did not potentiate the therapy (CI = 0.93, additive) (Figure 3F).

Siremadlin treatment was able to synergistically potentiate anti-PD-1 immunotherapy in the given doses with a CI of 0.72, an effect that was even more pronounced for the Sulanemadlin and anti-PD-1 immunotherapy group with a CI of 0.46 (Figure 3F). The increased delay in tumor doubling time as well as the observed synergistic effects of the combination treatments correlated with an increase in survival time (Figures 3G–3H). Here, the combination of anti-PD-1 immunotherapy and Sulanemadlin doubled the median survival time (50% survival) from 10 days in the control group to 20 days. The animals in the anti-PD-1 immunotherapy + Siremadlin treatment group displayed a heterogeneous response, while 50% of the animals discontinued the study by day 12.5, 2 individuals survived until the final endpoint (day 21) (Figure S5).

Hence, we conclude that *in vivo* treatment with the small molecule MDM2 inhibitor Siremadlin and the stapled peptide MDM2/MDMX inhibitor Sulanemadlin synergizes with anti-PD-1 treatment. Further, Sulanemadlin synergizes to a higher degree with anti-PD-1 treatment than Siremadlin, leading to an increase in survival time.

### Sulanemadlin treatment in combination with anti-PD-1 immunotherapy leads to enhanced tumor immunogenicity and increased tumor infiltrating lymphocytes *in vivo*

**Figure 4:**
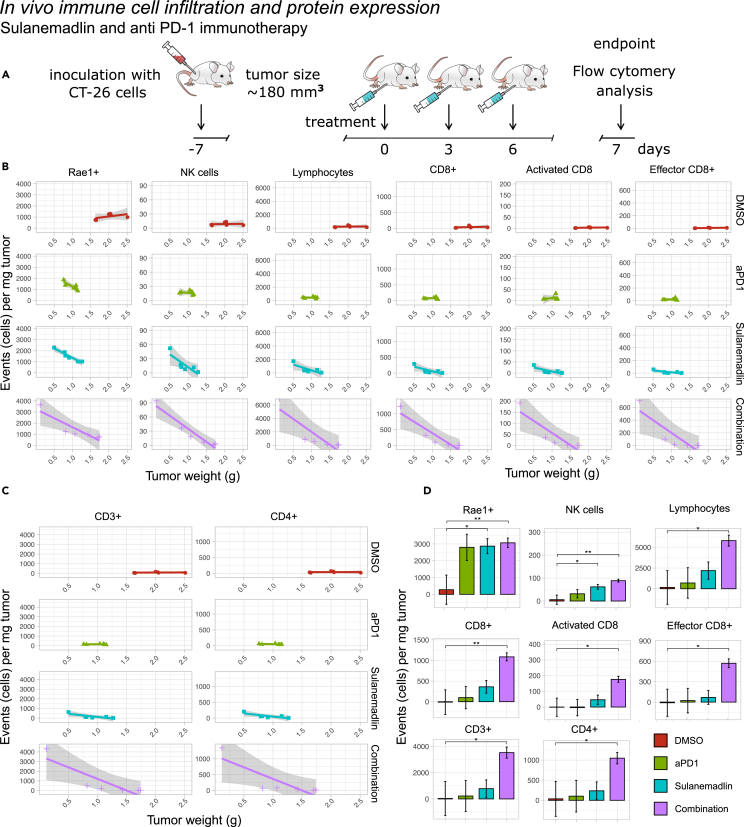
Sulanemadlin treatment in combination with anti-PD-1 immunotherapy leads to enhanced tumor immunogenicity and increased tumor infiltrating lymphocytes *in vivo* (A) Overview of the treatment schedule. BALB/c mice carrying CT26.WT tumors were treated with DMSO, Sulanemadlin (30 mg/kg, trans isomer) as well as anti-PD-1 immunotherapy (aPD-1) (5 mg/kg) in combination with Sulanemadlin (30 mg/kg). (B and C) Linear regression analysis of tumor tissue analyzed by flow cytometry revealed the relation between protein expression and tumor weight of RAE-1, NK cells, lymphocytes, and CD8^+^, activated CD8^+^ and effector CD8^+^ cells and (C) CD3^+^ and CD4^+^ cells. Debris, non-singlets and dead cells were excluded from the analysis. Line = polynomial fit, Gray field = confidence interval. There is a trend for increased cell counts by reduced tumor weight, indicating tumor infiltration. (D) Estimated marginal means of expression in low weight tumors, from a multivariable linear regression of tumor weight, expression and treatment modality (data are represented as estimated marginal means with standard error of the mean (SEM)). Significance was tested by comparing estimated slope for each linear regression over the entire range of tumor weights. ∗*p* < 0.05, ∗∗*p* < 0.01 indicates significantly different slope values of the linear regression between treatments, i.e., a significantly higher or lower tumor infiltration. See also Figures S6–S8.

Since the stapled peptide Sulanemadlin in combination with anti-PD-1 treatment appeared to be the most efficient combination to induce synergy, reduce tumor growth, and lead to increased survival, we next examined the effects on the immune system with these treatments. We used the clinical version of Sulanemadlin (*trans* isomer), as monotreatment and in combination with the anti-PD-1 treatment DX400. Mice were treated in the same manner as described for the therapy study, with intraperitoneal (i.p.) treatments every third day, but only until a total of three treatments was reached (summarized in Figure 4A). 24 h after the final treatment the tumors were harvested and stained for lymphocyte markers and analyzed by flow cytometry (Figures 4B–4D and S6–S8). The tumor weights were significantly smaller in all treatment groups compared to empty vehicle control, DMSO (Figure S7). However, there was a large variation in tumor size (weight) in the Sulanemadlin and anti-PD-1 immunotherapy combination group, with some tumors as large as those in the control group and some being among the smallest recorded tumors. When we analyzed the expression of RAE-1 in the CD45^−^tumor cells we noticed that the tumor size/weight correlated with RAE-1 protein expression, with a higher number of cells per mg of tumor expressing this marker the smaller the tumor was (Figure 4B). Therefore, we performed linear regression analysis of marker expression and tumor weight to search for significantly higher expression between treatments correlated to the therapy response. Both monotreatment with Sulanemadlin and the combination treatment, Sulanemadlin + anti-PD-1 immunotherapy led to a significantly higher number of RAE-1 positive cells/mg tumor as well as a significantly higher number of NK cells/mg tumor. (Figure 4B). This increase was not achieved with anti-PD-1 treatment alone. The number of tumor-infiltrating lymphocytes, CD3^+^, CD4^+^, CD8^+^, and CD8^+^ activated and effector T cells was also correlated with tumor size (Figures 4B–4D) with a significantly higher number of these cell populations per mg tumor in the tumors from mice treated with the Sulanemadlin + anti-PD-1 treatment combination (Figure 4D).

Furthermore, we examined the infiltration of naive CD4^+^ T cells, effector and memory CD4^+^ T cells, T regulatory cells, as well as mature B cells (Figure S8). Even here, we observed a significant increased infiltration per milligram of tumor in the responsive tumors of mature B cells, activated CD4^+^ T cells, CD4^+^ memory T cells, and T regulatory cells and a trend of increased infiltration for naive CD4^+^ T cells, effector CD4^+^ T cells, and CD8^+^ memory T cells, with the most notable increase observed in the combination therapy group.

Our results suggest that treatment with Sulanemadlin in combination with anti-PD-1 immunotherapy leads to a significantly higher influx of lymphocytes, especially CD8^+^ T cells, with more activated and effector cytotoxic T cells per mg tumor, correlating with an overall decreased tumor size after combination treatment compared to monotherapy.

## Discussion

Most successful cancer therapies are based on the use of combinations. Therefore, there is an intensive search for molecules that may cooperate with p53 stabilization, including the combination with radiation therapy, DNA-linking molecules, or CDK inhibition.^17^^,^^40^^,^^44^^,^^45^^,^^46^

In the recent years, the breakthrough of immunotherapy in cancer treatment has provided new hope for patients battling various types of cancer. However, challenges persist, including relatively low response rates and instances of hyperprogression.^47^ Efforts to identify predictive biomarkers for immunotherapy efficacy continue, with known markers such as PD-L1 expression, tumor mutational burden, microsatellite instability, and mismatch repair deficiency being investigated. Notably, MDM2 and MDMX amplification has emerged as a potential biomarker associated with hyperprogression during immunotherapy across various cancer types.^47^

Further, the success of immunotherapy in treating cancer has been limited to certain tumors characterized by the presence of cytotoxic lymphocytes.^31^ A strategy to broaden the use of immunotherapy would be to inhibit MDM2/X and enhance the immunogenicity of the tumor, increasing the infiltration of lymphocytes into tumors, reactivating the immune response. Previous studies have suggested that p53 activation could lead to enhanced immunogenicity and immune cell activity.^25^^,^^26^^,^^32^^,^^48^ However, little is known about how MDM2 and MDMX inhibition affects immune cells.

In this study, we investigated the effects of two small molecule MDM2 inhibitors and one stapled peptide MDM2/MDMX inhibitor. We found that *in vitro*, the small molecule MDM2 inhibitors Siremadlin and Navtemadlin inhibited tumor cell growth at lower concentrations than the stapled peptide Sulanemadlin. Both small molecules and stapled peptides induced a p53-dependent upregulation of PD-L1, indicating a possible benefit from an MDM2 inhibitor-immunotherapy combination. Interestingly, we found that the stapled peptide combined with anti-PD-1 treatment DX400 *in vivo* was more efficient in increasing survival than the anti-PD-1 + Siremadlin combination.

*In vitro*, murine CT26.WT epithelial cells responded to treatment very similarly to murine B16-F10 melanocytes. Both cell lines have a deletion in the CDKN2a gene encoding tumor suppressor proteins p19^ARF^ and p16^INK4A^, leading to silencing of wt p53.^49^^,^^50^ The human SJSA-1 osteosarcoma instead harbors an MDM2 amplification resulting in silencing of p53.^51^ It appears that human cell lines may have higher sensitivity compared to murine cell lines. This observation is consistent with the results of previous studies, which also found similar trends in the sensitivity of human versus murine cell lines to p53 therapy.^52^ Importantly, we clearly demonstrate that the growth-inhibiting effect from the compounds used in our study is completely dependent on the activation of p53, as B16-F10 p53^−/−^ cell growth was not affected by any of the treatments.

Cytotoxic CD8^+^ T cells recognize tumor neoantigens presented by the MHC I molecule (HLA in humans) of the tumor cells.^36^ Tumors with high mutational frequency display a high number of neoantigens presented by MHC I. These tumors typically have the highest immunogenicity.^53^ A strategy for tumors to evade the immune system is therefore to downregulate MHC I. The presence of MHC I is however an inhibitory signal for NK cells and the lack of MHC I may lead to NK cells attacking the tumor cells if these cells also express enough activating ligands.^38^ One such activating ligand is RAE-1 in mice.^39^ Interestingly, p53 has been suggested to be a regulator of MHC I and its peptide presentation.^54^^,^^55^ NK cell activating ligands UBLP1 and UBLP2 are also controlled by p53 activity.^56^ When we treated CT26.WT cells with Sulanemadlin *in vitro*, we observed an upregulation of p53, RAE-1, MHC I, and PD-L1 proteins. Navtemadlin also increased MHC I, PD-L1, and RAE-1 *in vitro* in the syngeneic but immunologically cold B16-F10 model. Upregulated MHC I protein suggests that neoantigen peptide presentation might be increased by MDM2 inhibition. This may be beneficial for cytotoxic T cell activation against the tumor. Since we noticed an upregulation of the NK cell activating ligand RAE-1 upon Sulanemadlin treatment *in vitro* and *in vivo*, there is a possibility of innate immune response activation in the CT26.WT model upon p53 activation. Additionally, Sulanemadlin treatment led to the induction of p21 and PUMA through p53 activation in CT26.WT cells, with p21 mediating G1 growth arrest and PUMA triggering apoptosis. We have previously shown that MDM2 inhibition as a monotreatment mainly leads to cell-cycle arrest but that pro-apoptotic proteins also are transcribed simultaneously as proteins promoting cell-cycle arrest.^40^ Likely, the amount and sustainability of the p53 protein expression controls the apoptotic threshold, as p53 needs to outbalance anti-apoptotic factors.^40^^,^^57^ The combined effect of increased MHC I and RAE-1 expression suggests that Sulanemadlin and small molecule MDM2 inhibitors may enhance tumor cell immunogenicity *in vivo*.

We chose to compare the small molecule inhibitor with the lowest IC_50_ value, Siremadlin, with the stapled peptide Sulanemadlin *in vivo*. We observed a significantly delayed growth from all treatments compared to the anti-PD-1 (DX400) isotype control or the inactive version of Sulanemadlin (non-binder). The treatment that led to the highest reduction in tumor doubling time and resulted in longest survival was the stapled peptide Sulanemadlin combined with anti-PD-1. Since Sulanemadlin is a large peptide and Siremadlin is a small molecule, the chosen dose at 30 mg/kg corresponded to around 3.6 times more Siremadlin molecules than Sulanemadlin peptides. Still, treatment with Sulanemadlin in combination with anti-PD-1 demonstrated enhanced efficacy *in vivo*, exhibiting a notable increase in synergistic effects and prolonging median survival. While Siremadlin at this concentration delayed tumor growth, it did not further extend median survival when combined with anti-PD-1 therapy. Variations in pharmacokinetic properties, including bioavailability, half-life, and tissue distribution, may underlie the differing potency observed. We hypothesize that disparities in the *in vivo* efficacies of small molecule MDM2 inhibitors and stapled peptide MDM2/MDMX may result from differences in their ability to sustain intracellular p53 activation. Notably, stapled peptides are engineered to resist proteasomal degradation, potentially enhancing their capacity to maintain prolonged intracellular p53 activity, which could contribute to their superior efficacy compared to small molecule inhibitors.^22^ Since MDM2 and MDMX function is largely dependent on their complex formation, there may be an advantage in inhibiting both MDM2 and MDMX. This highlights important differences between the efficacy of small molecule inhibitors and stapled peptides. These differences will be important to also determine in patients enrolled in clinical trials.

Here we chose to use the CT26.WT syngeneic model, since it has been previously reported to be partially responsive to treatment with PD-1 blocking antibodies.^34^ The current study demonstrates that activating p53 results in an enhanced effect of immune cell influx to tumors. We chose to investigate leukocyte populations in the tumors at a relatively early time point, after only three treatments. Since the size of the tumors corresponded nicely with the number of infiltrating immune cells in the tumor, it was clear that with more leukocytes infiltrating the tumor, tumor growth inhibition was promoted. MDM2 and p53 regulate the inflammatory signaling through regulation of the NF-κB inflammatory pathway.^58^ The MDM2-p53- NF-κB signaling is complex. However, it is known that tumor cells respond differently to MDM2 signaling depending on their basal levels of NF-κB.^59^ This suggests that the inflammatory profile of both tumor cells and immune cells may undergo alterations when p53 is activated through MDM2 inhibition. The activation of p53 through MDM2 inhibition can potentially disrupt the intricate balance of NFκB-mediated inflammatory signaling within the tumor microenvironment. In immune cells, however, p53 activation may modulate NF-κB activity in a manner that influences immune cell function and the inflammatory status.

Although the tumors from the mice treated with both Sulanemadlin and anti-PD-1 were heterogeneous in terms of size, the combination treatment led to more lymphocyte infiltration and higher number of CD3^+^ T cells and cytotoxic T cells per mg tumor. There were also more activated and effector CD8^+^ T cells per mg tumor in the combination treated mice. Monotreatment did not result in any significant changes in T cell infiltration. It has been previously reported that PD-1 inhibition does not impact the proliferation or activation of T cells. Rather, the production of cytokines such as IFNγ and degranulation is stimulated by anti-PD-1 treatment.^60^ Therefore, the noticed lack of effect in T cell number and activation status seen here by anti-PD-1 monotreatment, may be expected. In this study, increased infiltration of most CD45^+^ leukocytes corresponded to smaller tumor size, indicating the significance of the immune response for tumor eradication.

We detected an effect on the expression of RAE-1 upon treatment *in vitro* as well as *in vivo*, it is therefore likely that Sulanemadlin is capable of reaching the tumor efficiently. Interestingly, both the Sulanemadlin monotreatment and the combination treatment also led to an increased influx of NK cells into the tumors, suggesting that this RAE-1 expression has a functional impact. If the tumor microenvironment instead is inhibiting the immune response, the effect of Sulanemadlin treatment may differ between different tumor models and more importantly between patients, regardless of wt p53 status.

Our results carry significant implications for cancer treatment strategies. Both immunotherapy and p53 therapy have emerged as promising modalities in cancer treatment, with increasing interest in their combination. However, for this combined approach to be effective, it’s essential that p53 therapy does not compromise immune functions. Ideally, anti-cancer drugs should not only target tumors but also enhance anti-tumor immune responses. In a recent comprehensive pan-cancer study examining MDM2/X amplification across 250 studies involving 30,118 patients, it was found that amplification of MDM2/X expression predicts a poor response to immunotherapy and is associated with shorter overall survival.^47^ Therefore, inhibiting MDM2/X could potentially restore the efficacy of immunotherapy treatment. Our results suggest that early in treatment, the combination of Sulanemadlin and anti-PD-1 immunotherapy results in an influx of predominantly cytotoxic lymphocytes, which correlates with reduced tumor size. Furthermore, our findings indicate a distinct advantage in combining p53 therapy, especially MDM2/X inhibition, with anti-PD-1 treatment over time, as evidenced by a synergistic effect leading to more effective tumor growth suppression and prolonged survival in a model partially responsive to immunotherapy. This suggests that using Sulanemadlin in combination with anti-PD-1 treatment may be beneficial for patients with otherwise only partially immunologically responsive tumors.

### Limitations of the study

The presented study has several limitations. Due to supply constraints, both *trans* and mixed isomers of the stapled peptide were utilized. Variations in isomers may yield differing properties and effects, potentially impacting the accuracy and generalizability of study results. To prove the specificity of the treatment, we also attempted to delete the p53 gene from CT26.WT cells by CRISPR/Cas9 knockout techniques but were unable to achieve this result successfully. Additionally, conducting T cell or NK cell depletion experiments or employing CRISPR/Cas9 targeting of RAE-1, would provide valuable insights into the mechanisms underlying the effectiveness of the MDM2/X inhibition and PD-1 immunotherapy combination. These experiments could help elucidate how these immune cell populations contribute to the observed synergistic effects. Moreover, the *in vivo* therapy experiment is a first proof-of-concept study. To better understand the treatment’s efficacy, it is important to consider biological factors such as sex-related differences in response. Therefore, additional experiments including male subjects should be conducted to ensure a comprehensive evaluation. Additional dosages and treatment schedules should be tested and would also allow for more detailed and advanced synergy calculations.

## STAR★Methods

### Key resources table

**Table:** 

REAGENT or RESOURCE	SOURCE	IDENTIFIER
**Antibodies**		

Anti-p53 monoclonal rabbit anti-mouse (primary)	Abcam	Cat#ab246223
Goat anti-rabbit (secondary)	Dako	Cat#P0217; RRID:AB_2728719
Anti-MHC I/H-2Kb, PE-conjugated (clone AF6-88.5)	Biolegend	Cat# 116508; RRID:AB_313735
Anti-MHC I/H-2Kb, PerCP Cy5.5-conjugated (clone AF6-88.5)	Biolegend	Cat# 116516; RRID:AB_1967133
Anti-Pan RAE-1, PE-Vio 770-conjugated (clone REA723)	Miltenyi	Cat# 130-111-471; RRID:AB_2653326
Anti-B7-H1/PD-L1, BV711-conjugated (clone 10F.9G2)	Biolegend	Cat# 124319; RRID:AB_2563619
Anti-p53, AF 647-conjugated (clone 1C12)	Cell signaling technology	Cat# 2533; RRID:AB_2256294
Anti-p21, FITC-conjugated (clone F-5)	Santa Cruz Biotechnology	Cat#sc-6246 FITC
Anti-PUMAα, PE-conjugated (clone B-6)	Santa Cruz Biotechnology	Cat#sc-377015 PE
Anti-CD11b, BV510-conjugated (clone M1/70)	BD Biosciences	Cat# 562950; RRID:AB_2737913
Anti-CD45, BV786-conjugated (clone 30-F11)	BD Biosciences	Cat# 564225; RRID:AB_2716861
Anti-CD3, PerCPCy5.5-conjugated (clone 145-2C11)	eBioscience	Cat# 45-0031-82; RRID:AB_1107000
Anti-B220, PE-conjugated (clone RA3-6B2)	Biolegend	Cat#also 103208; RRID:AB_312992
Anti-CD8a, APC-conjugated (clone 53–6.7)	Biolegend	Cat#100712; RRID:AB_312750
Anti-CD4, BV605-conjugated (clone RM4-5)	Biolegend	Cat# 100548; RRID:AB_2563054
Anti-CD25, PECy7-conjugated (clone PC61)	BD Biosciences	Cat# 561780; RRID:AB_10893596
Anti-CD44, BV421-conjugated (clone IM7)	Biolegend	Cat#103040; RRID:AB_10895752
Anti-CD62L, BV711-conjugated (clone Mel-14)	Biolegend	Cat# 104445; RRID:AB_2564215
Anti-NKp46, FITC-conjugated (clone 29A1.4)	Biolegend	Cat#137606; RRID:AB_2149150
Anti-Foxp3, (clone FJK-16s)	eBioscience	Cat# 13-5773-82; RRID:AB_763540

**Chemicals, peptides, and recombinant proteins**		

Sulanemadlin (ALRN-6924 mixed isomer) (Ac-Leu-Thr-Phe-R8-Glu-Tyr-Trp-Ala- Gln-Leu-S5-Ala-Ala-Ala-Ala-Ala-DAla- NH2(olefin staple from R8 to S5))	Chinese peptides	Customized
Non-binder (ALRN-6924 F3 to dPhe) (Ac-Leu-Thr-DPhe-R8-Glu-Tyr-Trp-Ala- Gln-Leu-S5-Ala-Ala-Ala-Ala-Ala-DAla- NH2(olefin staple from R8 to S5))	Chinese peptides	Customized
Sulanemadlin (ALRN-6924 trans isomer)	DeliverTides	Cat#605054; CAS:1451199-98-6
Siremadlin (HDM201)	MedChemExpress	Cat#HY-18658/CS-7654; Cas:1448867-41-1
Navtemadlin (AMG 232, KRT 232)	Axon Medchem	Cat#2639; Cas: 1352066-68-2
mDX400	Merck & Co., Inc	DX400 D265A LPD2127/LPD2128
Isotype control: mIgG1	Merck & Co., Inc	TC31.27F11.C2

**Experimental models: Cell lines**		

Mouse: CT26.WT	ATCC	CRL-2638
Human: SJSA-1	ATCC	CRL-2098
Mouse: B16-F10	ATCC	CRL-6475
Mouse: B16-F10 p53−/− clone 8	Prepared inhouse from B16-F10	

**Experimental models: Organisms/strains**		

Mouse: BALB/cAnNRj	Janvier labs	Strain: BALB/cAnNRj

### Resource availability

#### Lead contact

Further information and requests for resources and reagents should be directed to and will be fulfilled by the lead contact, Dr. Diana Spiegelberg (diana.spiegelberg@uu.se).

#### Materials availability

This study did not generate new unique reagents.

#### Data and code availability

• All data reported in this paper will be shared by the lead contact upon request.
• This paper does not report original code.
• Any additional information required to reanalyze the data reported in this paper is available from the lead contact upon request.

### Experimental model and study participant details

#### Cell lines and cell culture

The murine cell lines CT26.WT (ATCC CRL-2638) (colon carcinoma, female), B16-F10 (CRL-6475) (malignant melanoma, male) and the human cell line SJSA-1 (CRL-2098) (osteosarcoma, male) were purchased from ATCC. At arrival, the cell lines were expanded and aliquots were frozen at low passage numbers. None of the cell lines were further authenticated. B16-F10 p53^−/−^(clone 8) was generated from the B16-F10 cell line,^40^ expanded and frozen.

Prior to experiments, the cell lines were thawed and allowed to adjust to culture for a minimum of one week, until normal growth rate was obtained. All cell lines were grown in RPMI-1640 medium (R8758 Sigma Aldrich) supplemented with 10% heat inactivated FBS (SV30160.03, Hyclone) and 100 U/mL penicillin and 100 μg/mL streptomycin (5140-122, Gibco). The cell lines were sub-cultivated before reaching confluency, at least twice per week. All cell cultures were maintained at 37°C and 5% CO_2_. None of the cell lines were maintained in culture for more than five months.

The cultures were tested and confirmed to be negative for mycoplasma infection every second month, using the MycoAlert Plus Detection kit (LT07-710, Lonza) according to the manufacturer’s instructions.

#### Animal studies/mice

For the survival study, female immunocompetent BALB/cAnNRj mice (*N* = 48, age = 6 weeks) were housed under standard laboratory conditions and fed *ad libitum*. 1 × 10^6^ CT-26.WT cells were injected subcutaneously in the right lower flank. Mice were divided randomly into the following groups (6 animals/group): a. Isotype control + HPβCD/DMSO, b. Isotype control + non-binder (ALRN-6924 F3 to dPhe), c. Isotype control + Sulanemadlin, d. Isotype control + Siremadlin, e. DX400 + HPβCD/DMSO, f. DX400 + non-binder (ALRN-6924 F3 to dPhe), g. DX400 + Sulanemadlin, h. DX400 + Siremadlin. When tumors reached an average size of 183 mm^3^, mice received MDM2/MDMX blocking peptides (30 mg/kg) and mAb’s (5 mg/kg) or vehicle every third day for up to twenty-one days. Tumor size was measured every third day using a digimatic caliper (Mitutoyo, Sweden) and the tumor volume was calculated as 4πabc/3 where a, b, and c were measured diameters in all dimensions. The combination effects of the treatment groups were assessed according to the BLISS independence method on day 6, when no animal had yet reached the endpoint. The combination Index (CI) was calculated as described earlier.^61^ In short: E_AB_ = E_A_ + E_B_(1-E_A_), where E_A_ and E_B_ represent the observed effects of drug A and B, and E_AB_ the effect of drug A combined with drug B. CI=(E_A_ + E_B_-E_A_E_B_)/E_AB_ being indicative of synergy (CI < 1), antagonism (CI > 1) or additivity (CI = 1).

For the tumor *in vivo* immune cell infiltration study; female immunocompetent BALB/cAnNRj mice (*N* = 24, age = 6–8 weeks) were inoculated with tumors as described above. Mice were randomly divided into groups of 6 and treated with DMSO control, Sulanemadlin 30 mg/kg, DX400 5 mg/kg, or co-treated with DX400 and Sulanemadlin starting 7 days after tumor inoculation, when tumors were small but palpable (drug formulations as described above). The mice were treated i.p. every third day for a total of three treatments. Animals were sacrificed 24 h after the last treatment, 14 days after tumor injections.

#### Blinding of experiments was not possible

All experiments complied with Swedish law and were performed with permission from the Uppsala Committee of Animal Research Ethics.

### Method details

#### Drug formulation and treatments

The stapled peptide Sulanemadlin (ALRN-6924 *trans*-isomer)(Aileron) was purchased from DeliverTides. The mixed isomer of ALRN-6924 and the ALRN-6924 (F3 to dPhe) (referred to as non-binder) were produced by Chinese Peptides. Small molecule MDM2 inhibitor Siremadlin (Novartis) was purchased from MedChemExpress (Catalog no: HY-18658/CS-7654). Navtemadlin (Kartos Therapeutics) was purchased from Axon Medchem, (Catalog no: 2639). Upon arrival, the compounds were dissolved in DMSO (D2650, Sigma-Aldrich) and frozen in aliquots.

For *in vitro* treatments, the dissolved compounds or DMSO alone (as empty vehicle control) were thawed and mixed with cell culture medium supplemented with FBS, Penicillin and Streptomycin (complete cell culture medium) to obtain the desired concentration. To explore the effect on cell growth of a range of concentrations of the compounds, serial dilutions were made in complete cell culture medium. In the case of serial dilutions or different treatment concentrations, the DMSO concentration corresponding to the highest concentration of compound was used as empty vehicle control, otherwise the DMSO concentration corresponded with the concentration of the compound used to treat the cells.

For *in vivo* treatments, Sulanemadlin and the non-binder (ALRN-6924 (F3 to dPhe)) were dissolved in DMSO at 30 mg/mL and administered i.p. in 30% (2-Hydroxypropyl)-β-cyclodextrin (HPβCD) (Sigma Aldrich, Merck, Germany) at 30 mg/kg. Siremadlin (in DMSO 50 mg/mL) was dissolved in 40% PEG300, 5% Tween-80, 50% saline administered i.p. at 30 mg/kg. Mouse modified DX400 mlgG1/Kappa and Isotype control mouse IgG1 D265A/Kappa were generously supplied by Merck & Co., Inc., (Rahway, NJ, USA) and dissolved in 20 nM Sodium Acetate 9% Sucrose, pH 5.5 and administered i.p. at 5 mg/kg.

#### Live cell imaging

To investigate the effect of treatment on cells in exponential growth, suitable seeding densities for all *in vitro* experiments were determined by seeding a variety of cell densities obtained by serial dilutions. The cells were seeded in 200 μL complete cell culture medium in flat-bottomed 96-well plates or in 2 mL complete cell culture medium in 6-well plates (TPP). The cell growth was monitored in the IncuCyte S3 (Sartorius) live cell imaging system for 120 h. The seeding densities resulting in confluent or close to confluent cells at 72–96 h were chosen for further live cell imaging experiments.

1 000 CT26.WT, B16-F10 p53+/+ and B16-F10 p53−/− cells and 2 000 SJSA-1 cells were seeded in 200 μL complete medium in each well in flat bottomed 96-well plates (TPP). The cells were allowed to attach and adjust to culture overnight at 37°C and 5% CO_2_. After 18–24 h in culture, 200 μL fresh complete cell culture medium containing indicated drugs was added to each well. The cells were photographed every second hour at 37°C and 5% CO_2_ in the IncuCyte S3 Live cell imaging system. 10X phase images were obtained and the confluency was calculated with the IncuCyte software. The IC_50_ values were calculated by normalizing all samples to DMSO control at 96 h.

All live cell imaging experiments were performed with each treatment condition in triplicate.

#### Western blotting

To assess changes in p53 expression levels after treatment with varying concentrations of MDM2 or MDM2/MDMX inhibitors, Western blotting was conducted on whole lysates obtained from CT26.WT and B16-F10 p53^+/+^, and B16-F10 p53^−/−^ cells. Cells were seeded (8 × 10^4^ cells in 4 mL/60 mm dish), allowed to attach for 24 h, and then treated with fresh medium containing DMSO, Navtemadlin, Sulanemadlin, or Siremadlin at indicated concentrations. Following 6 h treatment, lysates were collected in SDS lysis buffer (BioRad), heated for 5 min at 95°C, sonicated, and stored at −20°C until use. Protein concentrations were measured using the DC Protein Assay (BioRad) prior to electrophoresis of the lysates on a 4–15% polyacrylamide gel (15-well Mini-PROTEAN TGX gel, BioRad) using 1X Tris-Glycine SDS buffer (BioRad) for 50 min at 150 V. After stain-free activation of the gel for 5 min (ChemiDoc Touch, BioRad), proteins were transferred onto PVDF membranes using semi-dry transfer method (TurboBlot, BioRad) for 30 min and imaged to visualize total protein (ChemiDoc Touch, BioRad).^40^ Membranes were then blocked for 1 h using blocking buffer (5% milk in TBS containing 0.1% Tween 20), incubated overnight at 4°C with anti-p53 primary antibody (rabbit anti-mouse, Abcam, EPR20416-124; diluted 1:1000 in blocking buffer), washed using TBS containing 0.1% Tween 20, incubated for 1 h with HRP secondary antibody (goat anti-rabbit, Dako, diluted 1:2000), and washed again. Protein expression was visualized using chemiluminescence (Clarity Western ECL Substrate, BioRad).

#### Flow cytometry

For the *in vitro* immunogenicity experiments; 2 × 10^4^ CT26.WT cells were seeded in 2 mL complete cell culture medium/well in 6 well plates (TPP). Alternatively, 2 × 10^5^ CT26.WT or B16-F10 cells were seeded in 10 mL complete medium in 10 cm dishes. The cells were allowed to adhere overnight at 37°C and 5% CO_2_ before treatment was initiated. Sulanemadlin (*trans*-isomer), Siremadlin or Navtemadlin, at indicated concentrations, was added in fresh complete cell culture medium, DMSO and non-binder were used as controls. After 48 h of treatment at 37°C and 5% CO_2_, cells were washed with PBS (D8537, Sigma-Aldrich)) and CT26.WT cells were mechanically detached by scraping while B16-F10 were detached using enzyme-free, PBS based cell dissociation buffer (13151-014, Gibco). Samples were plated in a V-bottomed 96-well plate (BR781601, Sigma Aldrich) prior to staining. To avoid unspecific antibody binding, cells were first Fc blocked 1:400 (2.4 G2, BD Biosciences) and dead cells were stained with Live/Dead Fixable Aqua Dead Cell Stain Kit 1:400 (L34966, Invitrogen) in PBS for 10 min at room temperature, protected from light. The cells were washed with 1% heat-inactivated FBS and 2 mmol/L EDTA in PBS (FACS buffer). Cells were then stained extracellularly for 30 min on ice, in FACS buffer, as in Table S2. The cells were washed in FACS buffer, fixed for a minimum of 30 min and a maximum of 18 h at 4°C and intracellularly stained with the eBioscience Foxp3/Transcription Factor Staining Buffer Set (00-5523-00 Invitrogen) according to manufacturer’s instructions. The intracellular antibody cocktail was mixed as in Table S2. After 30–60 min staining on ice, the samples were washed and resuspended in FACS buffer. Single stained controls were used for compensations. The samples were acquired in a BD LSR II or in a CytoFLEX LX (Beckman Coulter) flow cytometer and the data was analyzed using FlowJo version 10 (Treestar).

For the tumor *in vivo* immune cell infiltration study; tumors were excised at endpoint, 24 h after the last treatment. All tumors were weighed and then placed on ice in DMEM (D6429, Sigma-Aldrich) supplemented with 2% heat-inactivated FBS (SV30160.03, Hyclone), 1 × MEM amino acids (11130-036, Gibco), 1 × MEM Non-Essential Amino Acids (11140-050, Gibco),15 mmol/L HEPES (15630-056, Gibco), 150 μg/mL Liberase (05401127001, Roche) and 100 μg/mL DNAse I (11284932001, Roche). The tissues were mechanically dissociated using scissors and then incubated for 30 min at 37°C to activate enzymatic dissociation. The samples were incubated on ice for an additional 10 min. All samples were filtered and further dissociated through 70 μm strainers and washed with wash buffer: PBS supplemented with 0.5% BSA (A9647, Sigma-Aldrich), 2 mmol/L EDTA (E177, Amresco), 4.5 g/L dextrose (G8270, Sigma-Aldrich), 2 mmol/L L-glutamine (25030081, Gibco), 1 × MEM Amino acids (11130-036, Gibco), 1 × MEM Non-Essential Amino Acids (11140-050, Gibco) and 1 mmol/L Sodium Pyruvate (11360-070, Gibco). Red blood cells were lysed at room temperature for 4 min with 1X red cell lysis buffer (420302, Biolegend) and then washed in wash buffer. The samples were plated in V-bottomed 96-well plates (BR781601, Sigma Aldrich) for staining. Non-specific antibody binding was prevented by incubation with Fc block 1:400 (2.4 G2, BD Biosciences) and dead cells were stained with Live/Dead Fixable Aqua Dead Cell Stain Kit 1:400 (L34966, Invitrogen) in PBS for 10 min. The samples were extracellularly stained for 30 min with antibody cocktails in FACS buffer, as in Tables S3 and S4. All samples were then washed in FACS buffer and fixed overnight (for 11 h) at 4°C and intracellularly stained using the eBioscience Foxp3/Transcription Factor Staining Buffer Set (00-5523-00 Invitrogen) according to manufacturers instructions. Intracellular antibody cocktails are listed in Table S4. Compensations were done using single stained controls. The samples were acquired in a BD LSR II flow cytometer and the data was analyzed using FlowJo version 10 (Treestar).

### Quantification and statistical analysis

If not otherwise stated, a One-way ANOVA statistical analysis, followed by Tukey’s post hoc test was performed using the GraphPad Prism software, version 10.

The tumor doubling time was calculated using the modified Schwartz formula: tumor doubling time = [ln2 × ΔT]/[ln (X2/X1)], X1 = the tumor size at day 0, initial treatment day (average tumor size 100 mm^3^), X2 = tumor size at day 6 (last day before some animals reached endpoint (2000 mm^3^), ΔT = time (in days) between the two measurements.

Detected events (cells) per mg tissue for each of the markers on the flow cytometry panel were analyzed using a multiple linear regression model, with marker, treatment type and tumor weight as factors, as a result of the clear linear association between tumor weight and marker expression seen in the data. Marker expression between treatments and/or control were considered decreased or increased if the estimated marginal means from the linear model were significantly different (*p* < 0.05). Tests of homoscedasticity and normality were performed for model validation. R version 4.2.2 with RStudio version 2022.07.03 was used for the analysis.

## Supplemental information

Document S1. Figures S1‒S8, Tables S1, and S2
