# Supplementary material for: MDM2/MDMX inhibition by Sulanemadlin synergizes with anti-Programmed Death 1 immunotherapy in wild-type p53 tumors
Source: iScience. 2024 May 6;27(6):109862. doi: 10.1016/j.isci.2024.109862 (PMC11112618; doi:10.1016/j.isci.2024.109862)
Supplement: Document S1. Figures S1‒S8, Tables S1, and S2 [file mmc1.pdf]

iScience, Volume *27*

Supplemental information

MDM2/MDMX inhibition by Sulanemadlin synergizes with anti-Programmed Death 1 immunotherapy in wild-type p53 tumors

Katrine Ingelshed, Marit M. Melssen, Pavitra Kannan, Arun Chandramohan, Anthony W. Partridge, Long Jiang, Fredrik Wermeling, David P. Lane, Marika Nestor, and Diana Spiegelberg

Supplementary Figure 1

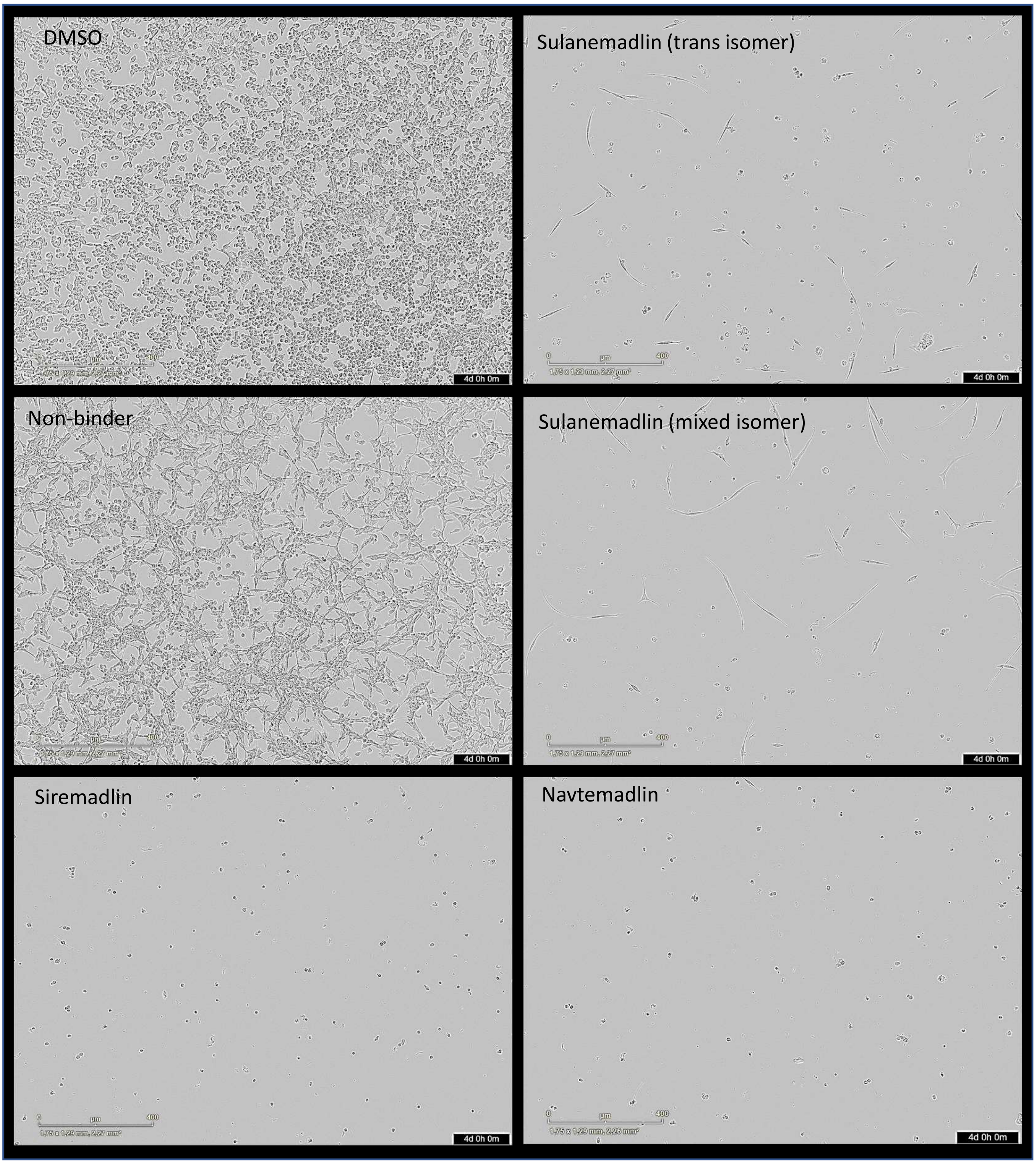

Supplementary Figure 1: Treatment at high concentration leads to cell death, related to Figure 1. Treatment of CT26.WT cells at the highest tested concentration, 16µmol/L, for 96 hours with indicated compounds results in cell debris or cell debris together with a few elongated cells. Representative images from Incucyte S3 live cell imaging.

Supplementary Figure 2

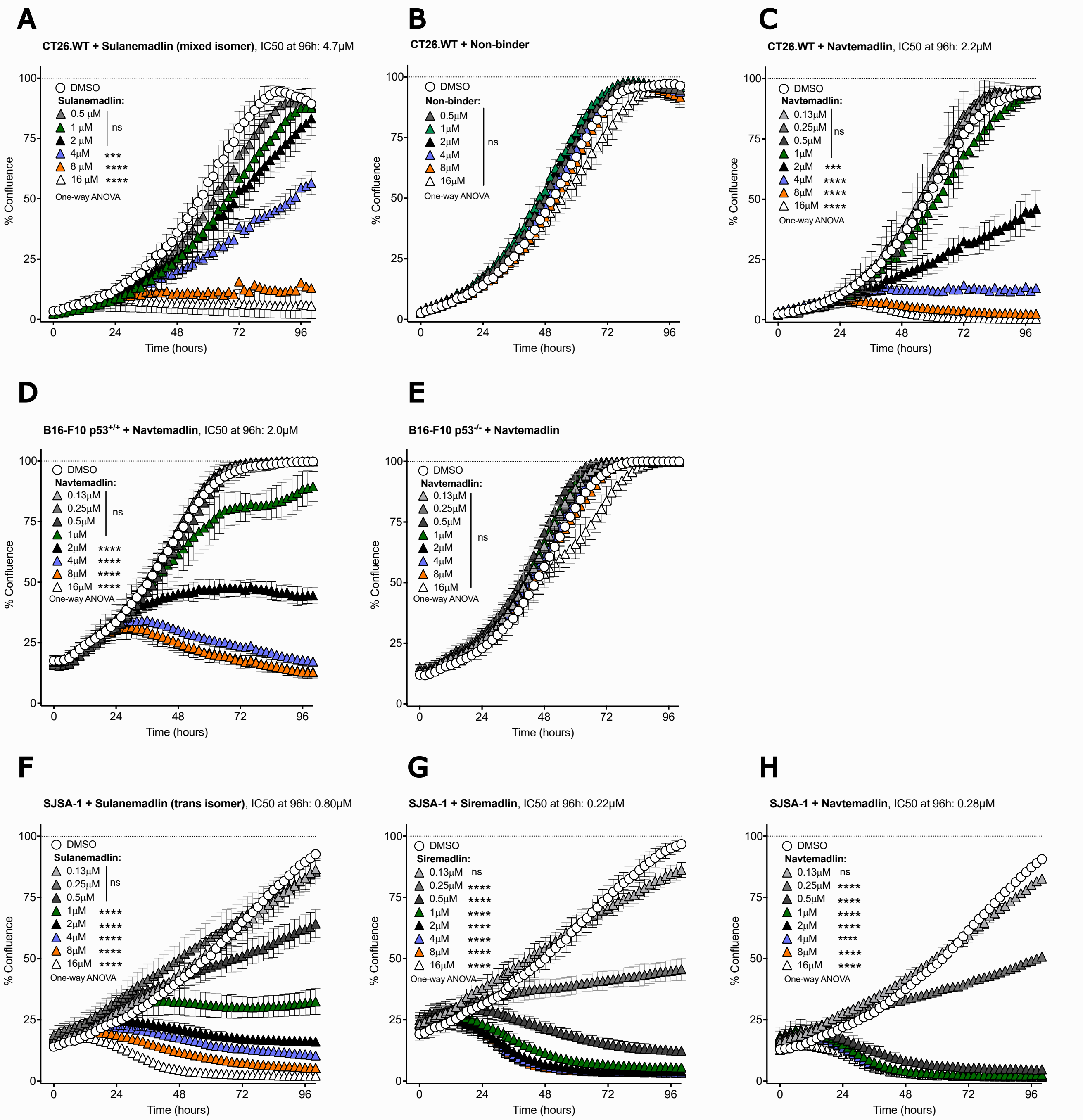

Supplementary Figure 2: MDM2 and MDMX inhibition prevent cancer cell growth p53 dependently *in vitro*, related to Figure 1. A-H: CT26.WT murine colon carcinoma cells, B16-F10 p53+/+ and B16-F10 p53-/- murine malignant melanoma cells and human SJSA-1 osteosarcoma cells were treated with indicated concentrations of Sulanemadlin (mixed isomer), Non-binder, Navtemadlin, Siremadlin or Sulanemadlin (trans isomer) and cell growth was monitored in the Incucyte S3 live cell imaging system. Proliferation was calculated as % of confluence over time. The IC50 value was normalized to DMSO control and calculated after 96 hours treatment. Representative data from at least three experiments performed in triplicates and a mean IC50 from all performed experiments are shown. *** P < 0.001; **** P < 0.0001. Oneway ANOVA, (mean, SD).

Supplementary Figure 3

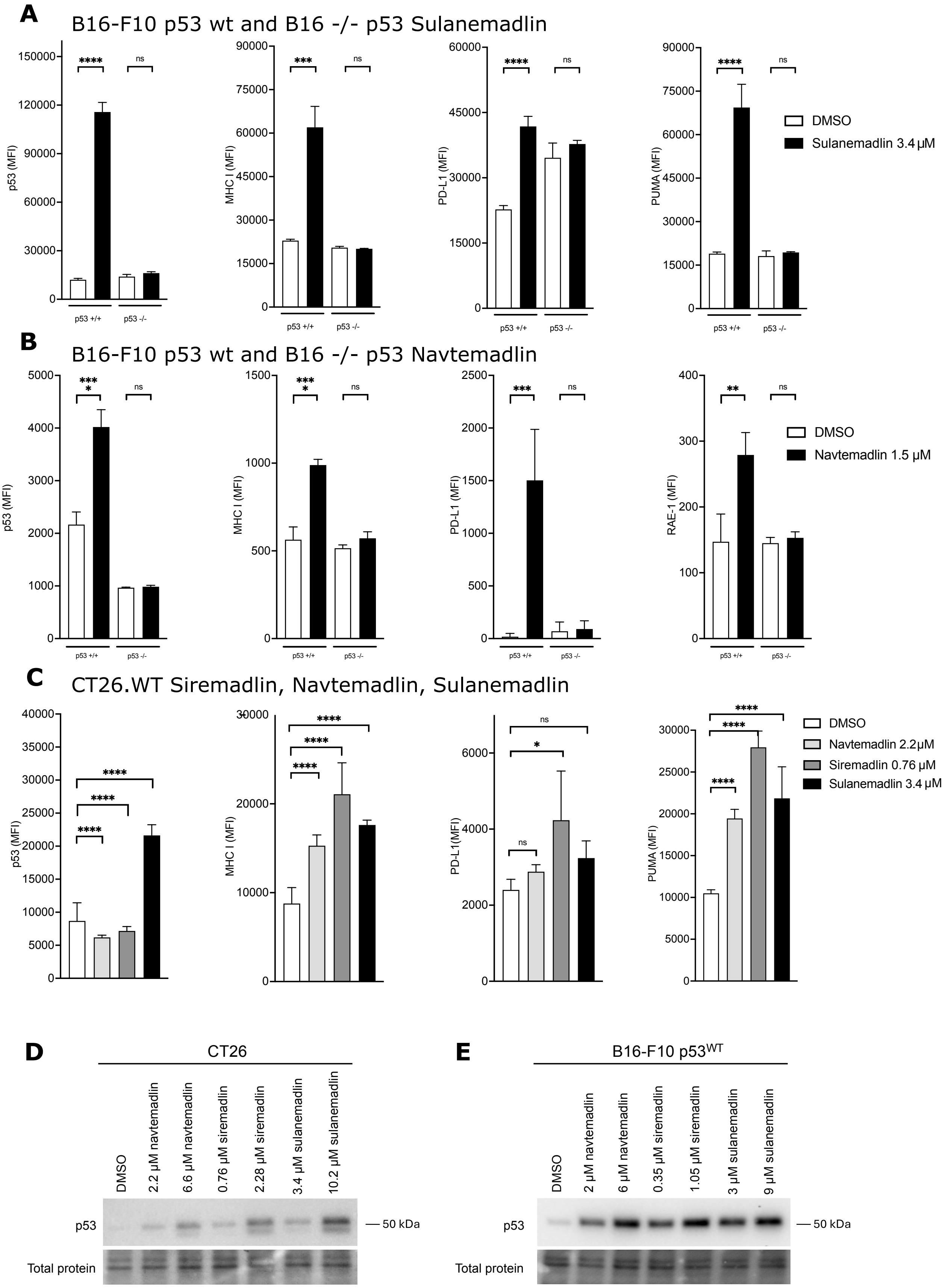

Supplementary Figure 3: Activation of p53 leads to increased expression of immunogenicity markers, related to Figure 2. Flow cytometry analysis of B16-F10 p53 wt and p53 KO murine malignant melanoma cells treated with A Sulanemadlin and B Navtemadlin of immune marker and p53 downstream target protein expression measured as median fluorescent intensity (MFI). C Flow cytometry analysis of murine CT26.WT cells treated Sulanemadlin, Siremadlin and Navtemadlin of immune marker and p53 downstream target protein expression measured as MFI. Fluorescence minus one (FMO) controls were used to detect and exclude background. Gating excluded debris and dead cells and only included single cells. The data are representative of at least two experiments. **P <0.01; ***P <0.001 **** P < 0.0001 One-way ANOVA, (mean with SD). Levels of p53 protein detected by Western blotting in D CT26.WT and E B16-F10 cells treated with two different concentrations of Sulanemadlin, Siremadlin and Navtemadlin for 6 hours. Representative immunoblots and total protein images are shown from one biological experiment. Images are cropped from full-length blots, which are shown in Supplementary Figure 4. .

Supplementary Figure 4

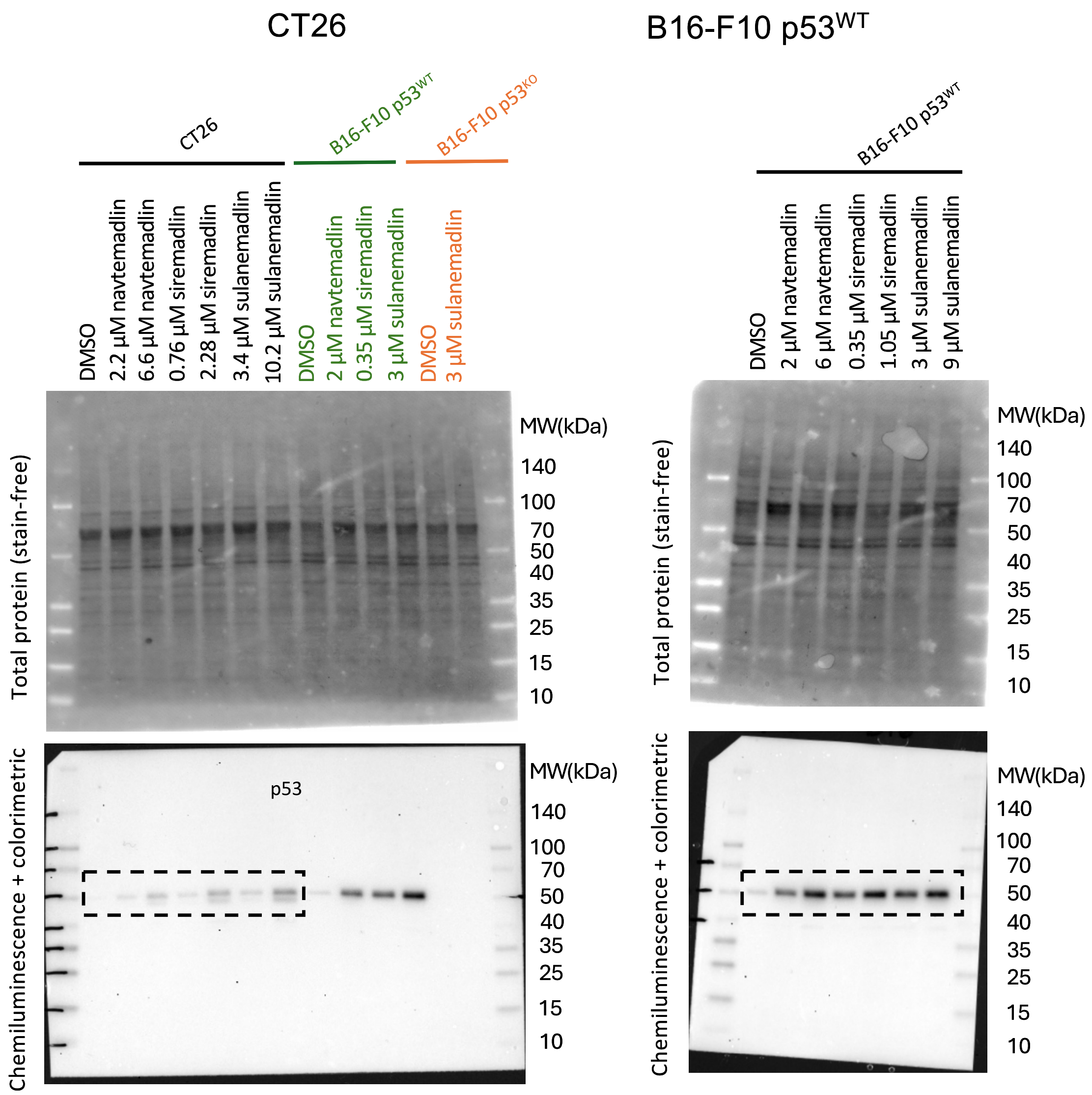

Supplementary Figure 4: Full-length blots from SI Figure 3, related to Figure 2, depicting total protein (stain-free membrane, upper panel) and p53 antibody staining (chemiluminescence and colorimetric merged, lower panel) in CT26.WT and B16-F10 cell lines. .

Supplementary Figure 5

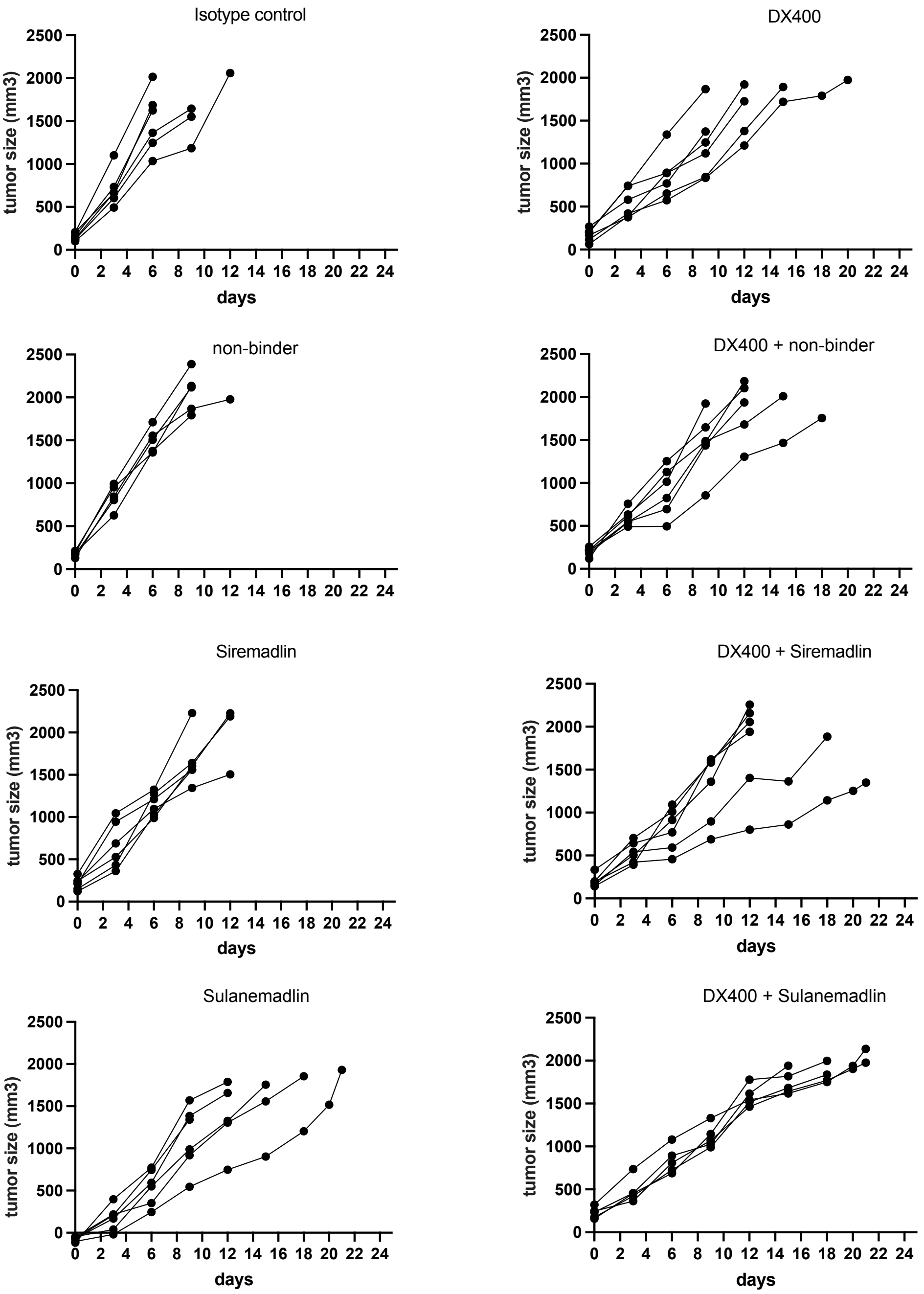

Supplementary Figure 5: *In vivo* treatment with Sulanemadlin or Siremadlin in combination with PD-1 inhibitor DX400 leads to reduced tumor sizes, related to Figure 3. BALB/c mice with CT26.WT tumors were monitored and tumors were measured every third day. Individual tumor sizes over time shown for the indicated treatment groups. .

Supplementary Figure 6

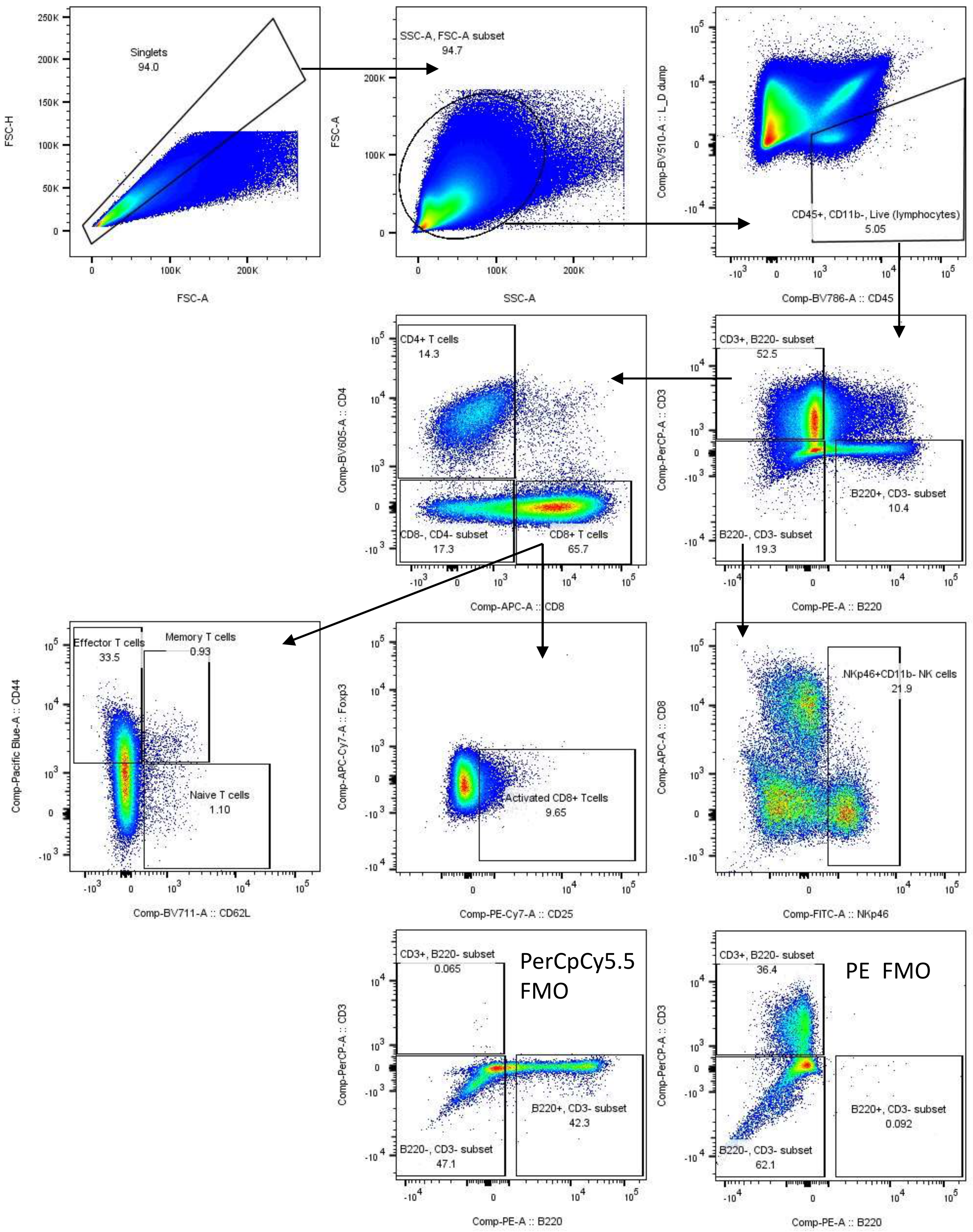

Supplementary Figure 6: Gating strategies for the analysis of tumor infiltrating lymphocytes, related to Figure 4. Single cells were gated according to size. Debris, noise and the most granular cells were excluded in the SSC-A, FSC-A gating. Lymphocytes (CD45+CD11b-), NK cells (CD45+CD11b-CD3-B220-NKp46+), T cells (CD45+CD11b-B220-CD3+) and T cell subsets (gating as indicated). FMO controls were used to define the gates. Dead cells and CD11b+ cells were both stained with BV510 and excluded in the lymphocyte gating.

Supplementary Figure 7

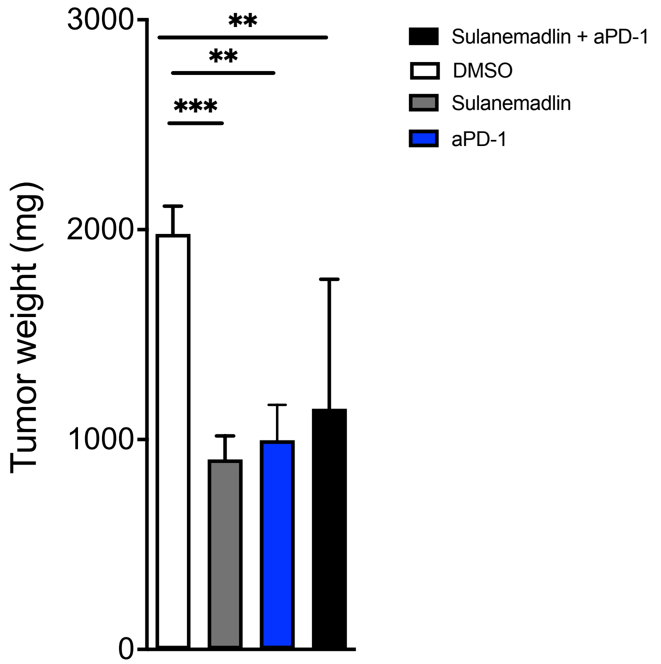

Supplementary Figure 7: Tumor weights, related to Figure 4. Tumor weights 14 days after tumor injections and after a total of three treatments. **P <0.01; ***P <0.001 One-way ANOVA, (mean with SD).

Supplementary Figure 8

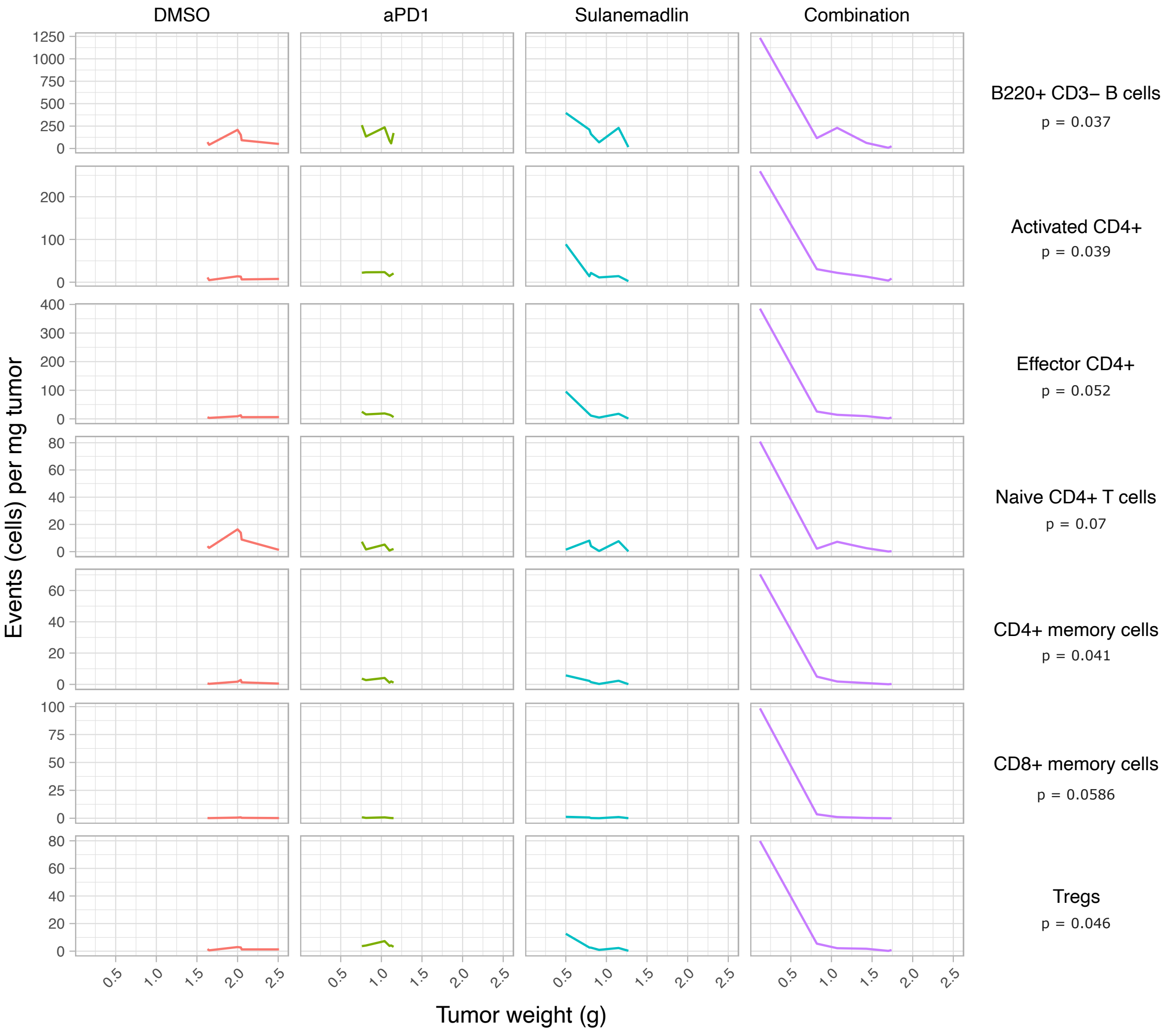

Supplementary Figure 8: Tumor size (weight) correlates with increased immune cell infiltration, related to Figure 4. Linear regression analysis of tumor tissue analyzed by flow cytometry revealed the relation between immune cell infiltration and tumor weight. Line = polynomial fit. There is a trend for increased cell counts by reduced tumor weight, indicating tumor infiltration. Significance was tested by comparing estimated slope for each linear regression over the entire range of tumor weights. P values indicate different slope values of the linear regression between DMSO and Combination treatments. No statistically significant difference was observed between the other groups.

Supplementary Table 1

treatment	tumor doubling time (days)	p value	summary
5 mg/kg isotype control	1.83	-	-
30 mg/kg non-binder	1.91	> 0.9999	ns
5 mg/kg PD-1 therapy (DX400)	2.72	0.2590	ns
30 mg/kg Sulanemadlin	2.60	0.2775	ns
30 mg/kg Siremadlin	2.48	0.5973	ns
5 mg/kg DX400 + 30 mg/kg non-binder	2.75	0.0672	ns
5 mg/kg DX400 + 30mg/kg Sulanemadlin	3.42	0.0022	**
5 mg/kg DX400 + 30mg/kg Siremadlin	2.97	0.0154	*

Supplementary table 1: Tumor doubling time, related to Figure 3. Tumor doubling time in BALB/c mice inoculated with CT26.WT tumors and treated with monotherapy or combination therapy. *P <0.05, **P <0.01 One-way ANOVA.

Supplementary Table 2

	Marker	Fluorophore	Dilution	Clone	Source
Extra-cellular	MHC I/H-2Kb	PE	1:50	AF6-88.5	Biolegend
	MHC I/H-2Kb	PerCP Cy5.5	1:50	AF6-88.5	Biolegend
	Pan RAE-1	PE-Vio 770	1:100	REA 723	Miltenyi
	B7-H1/PD-L1	BV711	1:50	10F.9G2	Biolegend
Intra-cellular	p53	AF 647	1:75	1C12	Cell signaling Technology
	p21	AF 488	1:100	F-5	Santa Cruz Biotechnology
	PUMAα	PE	1:100	B-6	Santa Cruz Biotechnology

Supplementary table 2: Antibody panel used to stain *in vitro* treated cells, related to Figure 2 and SI Figure 3. Single cell suspensions were first stained with an extracellular antibody cocktail. After fixation cells were stained with the intracellular cocktail. When B16-F10 cells were not stained for PUMA, the cells were stained with the PE-conjugated MHC I antibody. For all other experiments, the PerCP Cy5.5 conjugated MHC I antibody was used.

Supplementary Table 3

Marker	Fluorophore	Dilution	Clone	Source
RAE-1	PEVio770	1:100	RAE 723	Miltenyi Biotec
CD45	BV 786	1:300	30-F11	BD Biosciences

Supplementary table 3: Antibodies used for the flow cytometric *in vivo* analysis of tumor cells, related to Figure 4. Tumor cells were stained with the indicated cocktail of extracellular antibodies. .

Supplementary Table 4

	Marker	Fluorophore	Dilution	Clone	Source
Extra-cellular	CD11b	BV510	1:400	M1/70	BD Biosciences
	CD45	BV 786	1:300	30-F11	BD Biosciences
	CD3	PerCPCy5.5	1:50	145-2C11	eBioscience
	B220	PE	1:100	RA3-6B2	Biolegend
	CD8a	APC	1:200	53-6.7	Biolegend
	CD4	BV605	1:200	RM4-5	Biolegend
	CD25	PECy7	1:200	PC61	BD Biosciences
	CD44	BV421	1:200	IM7	Biolegend
	CD62L	BV711	1:200	Mel-14	Biolegend
	NKp46	FITC	1:200	29A1.4	Biolegend
Intra-cellular	Foxp3		1:100	FJK-16s	Invitrogen
	Streptavidin	APC/Fire750	1:100	Catalog 405250	Biolegend

Supplementary table 4: Antibody panel used to stain tumor infiltrating lymphocytes, related to Figure 4. Single cell suspensions from tumors were stained with an extracellular antibody cocktail, fixed and stained with intracellular antibody.
